# Effects of Bentonite, Zeolite, and Illite as Dietary Supplements for Pacific White Shrimp (*Litopenaeus vannamei*)

**DOI:** 10.3390/biology14121691

**Published:** 2025-11-27

**Authors:** Soohwan Kim, Seong-Mok Jeong, Hyun Mi Jung, Sara Lee, Seunghan Lee, Hyon-Sob Han

**Affiliations:** 1 West Sea Fisheries Research Institute, National Institute of Fisheries Science, Incheon 22383, Republic of Korea; penaeus@korea.kr (S.K.); jeonghm@korea.kr (H.M.J.);; 2 Aquafeed Research Center, National Institute of Fisheries Science, Pohang 37517, Republic of Korea; smjeong1@korea.kr; 3 Department of Aquaculture and Aquatic Science, Kunsan National University, Gunsan 54150, Republic of Korea; smartfish@kunsan.ac.kr

**Keywords:** Shrimp aquaculture, protein digestibility, functional feed additive, feed utilization, antioxidant status

## Abstract

This study evaluated the effects of dietary supplementation with three silicate minerals (bentonite, zeolite, and illite) on the growth performance, whole-body composition, hematological and immune parameters, and apparent digestibility of Pacific white shrimp (*Litopenaeus vannamei*). Four isonitrogenous (35% crude protein) and isoenergetic (17.0 MJ kg^−1^) diets were formulated: a control (CON) and three experimental diets containing 5% bentonite (BE), zeolite (ZE), or illite (IL), in which an equivalent amount of starch was replaced. Juvenile shrimp specimens (initial weight: 0.02 g) were reared for nine weeks and assessed for growth performance, feed efficiency, survival, biochemical indices, immune responses, and apparent digestibility. Shrimp specimens fed the illite-supplemented diet exhibited significantly higher final body weight (5.95 ± 0.97 g), weight gain, specific growth rate, and protein efficiency ratio than the other groups (*p* < 0.05). The feed conversion ratios were significantly better in the IL (1.10 ± 0.03) and ZE (1.16 ± 0.02) groups than that in the BE (1.26 ± 0.05) and CON (1.32 ± 0.04) groups (*p* < 0.05). The whole-body crude protein content was significantly higher in the ZE and IL groups. Hemolymph glutamic-oxaloacetic transaminase (GOT) and glutamic-pyruvate transaminase (GPT) activities were significantly reduced in the ZE and IL groups, indicating improved hepatic health. Non-specific immune responses, including nitro blue tetrazolium (NBT), phenoloxidase (PO), glutathione peroxidase (GPx), and lysozyme activities, were most enhanced in shrimp fed the IL diet. Apparent crude protein digestibility peaked in the IL group (93.3 ± 0.70%) followed sequentially by the CON (87.3 ± 0.92%), BE (87.8 ± 0.88%), and ZE (89.1 ± 1.11%) groups (*p* < 0.05). These findings demonstrate that dietary supplementation with illite significantly improves the growth performance, feed efficiency, hepatic condition, innate immune response, and protein digestibility of *L. vannamei*. Illite appears to be a promising candidate for functional feed supplementation aimed at enhancing productivity and health in intensive shrimp aquaculture.

## Simple Summary

Shrimp farming is a major part of aquaculture, but maintaining shrimp health and maximizing growth are ongoing challenges. In this study, we tested three natural minerals—bentonite, zeolite, and illite—as feed additives to improve the health and growth of Pacific white shrimp (*Litopenaeus vannamei*). These minerals are known to help with digestion and immunity in animals. Among the three, illite showed the most positive effects. Shrimp fed diets containing illite grew faster, digested protein more efficiently, and showed stronger immune responses. They also had better liver health, which is important for overall survival and performance. Our findings suggest that illite can be used as a functional and eco-friendly feed additive to support healthy and productive shrimp farming.

## 1. Introduction

The Pacific white shrimp (*Litopenaeus vannamei*) is an important species in global aquaculture because of its rapid growth rate, euryhaline tolerance, high fecundity, and adaptability to intensive production systems [1]. In 2022, *L. vannamei* accounted for more than 6.8 million metric tons of global farmed shrimp production, representing nearly 80% of the total output [1]. This dominance reflects the effectiveness of selective breeding programs, development of pathogen-resistant genetic lines, and major advances in feed formulation and water quality management, particularly in leading producing countries, such as China, India, Vietnam, and Ecuador [1,2].

Despite these advancements, concerns regarding the long-term sustainability of *L. vannamei* aquaculture have persisted due to biological, environmental, and regulatory pressures. Disease outbreaks caused by *Vibrio* spp., such as acute hepatopancreatic necrosis disease (AHPND), have persisted as a major source of economic loss in regions, including Asia and Latin America [3]. Additionally, the widespread use of antibiotics has raised global concerns regarding antimicrobial resistance, residual accumulation in shrimp tissues, and export restrictions in key markets, such as the European Union and North America [4,5]. In response to these challenges, increasing attention has been directed toward natural dietary supplements regarded as safe and sustainable for improving growth, supporting immune function, and mitigating environmental stress without reliance on synthetic chemicals [6]. Minerals such as bentonite (BE), zeolite (ZE), and illite (IL) are increasingly used as functional feed additives owing to their strong adsorptive and ion-exchange capacities and ancillary pH-buffering effects [7,8]. In aquafeeds, these properties are hypothesized to indirectly support growth and health by stabilizing the gastrointestinal milieu (e.g., reducing luminal irritants and fluctuation) and improving nutrient utilization, rather than exerting direct immunostimulatory effects [9,10].

BE, a swelling aluminosilicate predominantly composed of montmorillonite, possesses a large surface area and net negative charge, making it effective for binding mycotoxins, heavy metals, and bacterial toxins [11,12]. Its incorporation into aquafeeds has yielded improvements in growth performance, feed efficiency, antioxidant status, and organ health in fish species, such as European sea bass (*Dicentrarchus labrax*) [8]. ZE, especially the clinoptilolite variant, is a microporous aluminosilicate that exhibits strong cation-exchange and ammonia-binding properties. Dietary supplementation with ZE has shown advantages in nitrogen excretion, toxic metabolite suppression, feed conversion ratio (FCR) enhancement, and non-specific immunity upregulation in shrimp and fish [13,14].

IL, a non-swelling 2:1 phyllosilicate clay mineral with a characteristic layered aluminosilicate structure, is abundant in regions such as Yeongdong-gun, Republic of Korea, and has long been utilized as a functional mineral in livestock feed [15,16,17]. In ruminants, dietary IL has been shown to modulate rumen fermentation patterns, shift microbial community structures, and markedly reduce methane emissions under high-concentrate feeding regimes, reflecting its broad metabolic influence [17]. Similarly, in swine and poultry, IL supplementation improved growth performance, nutrient digestibility, carcass and meat quality, intestinal microflora balance, and egg production, with some responses being further enhanced when combined with probiotics [15,16]. Supplementation with clay minerals that included illite improved growth performance, nutrient digestibility, ileal villus height, footpad condition, and cecal and fecal microbial profiles under cyclic heat-stress conditions [18]. Furthermore, spraying illite and zeolite directly onto broiler litter enhanced litter quality, significantly reduced pathogenic bacteria such as Escherichia coli and Salmonella spp., and decreased footpad dermatitis scores, supporting the potential of illite-based materials to improve both intestinal health and environmental hygiene in intensive production systems [19]. Together, these findings highlight the multifunctional physiological properties of IL across terrestrial species, including its roles in nutrient absorption, gastrointestinal modulation, microbial balance, and detoxification.

In contrast, although BE and ZE have been widely examined across multiple aquaculture species, including shrimp, the application of pure IL in shrimp aquaculture remains highly limited. A recent study in African catfish evaluated a mixed-layer montmorillonite–illite/muscovite mineral rather than pure IL and reported modest improvements in growth uniformity and welfare indicators such as reduced skin lesions and stable hematological responses, demonstrating the physiological compatibility of illite-based minerals in fish [20]. However, to date, no studies have assessed the direct dietary use of pure IL in penaeid shrimp, indicating a substantial knowledge gap in the application of IL as a functional mineral in shrimp aquafeeds. Therefore, the present study was designed to investigate and compare the effects of dietary supplementation with BE, ZE, and IL on growth performance, hematological indices, immune responses, and apparent digestibility in *L. vannamei*.

## 2. Materials and Methods

### 2.1. Preparation of Experimental Diets

Four isonitrogenous and isoenergetic experimental diets were formulated to contain approximately 35% crude protein and approximately 9.7% crude lipid, yielding an estimated gross energy value of 17.0 MJ/kg. All diets were produced at Kunsan National University. The control diet (CON) was formulated using 40% tuna by product meal, 20% squid liver powder, 15% soybean meal, and 15% wheat flour as the primary protein and carbohydrate sources. Additionally, 3% fish oil, 1% mineral premix, and 1% vitamin premix were incorporated. In the three experimental diets, 5% of either BE, ZE, or IL was added to the basal formulation by replacing an equivalent amount (5%) of starch in the CON. We selected a 5% inclusion level for bentonite, zeolite, or illite by replacing starch in an isonitrogenous/isoenergetic formulation because this pragmatic dose is commonly employed for silicate minerals, allows detectable responses in growth and digestibility, and avoids nutrient-dilution or pellet-quality issues associated with higher inclusions. This resulted in four dietary treatments: CON, BE, ZE, and IL (Table 1). The three commercial minerals used in this study—bentonite (BE), zeolite (ZE), and illite (IL)—were characterized based on manufacturer specifications and commonly reported values for Korean commercial-grade mineral additives. All minerals were supplied in fine powdered form (particle size < 100 μm) for diet preparation. BE was a montmorillonite-based bentonite with a typical oxide composition of SiO_2_ 55–65%, Al_2_O_3_ 15–20%, MgO 2–5%, and Fe_2_O_3_ 2–4%. The particle size range was approximately 75–100 μm, and the specific surface area was 60–80 m^2^/g. ZE was a clinoptilolite-type zeolite with a three-dimensional porous crystalline aluminosilicate framework. Its commercial composition commonly includes SiO_2_ 60–70% and Al_2_O_3_ 12–15%, with minor amounts of K_2_O, CaO, and MgO. Particle size was below 100 μm, and the specific surface area was 20–40 m^2^/g. IL was a non-swelling 2:1 phyllosilicate clay mineral with a layered aluminosilicate structure. Commercial IL typically contains SiO_2_ 45–55%, Al_2_O_3_ 20–30%, and K_2_O 4–8%, with trace levels of MgO and Fe_2_O_3_. The IL used in this study consisted of particles < 100 μm, and its specific surface area was 10–25 m^2^/g. All dry ingredients were finely ground into a powder and homogeneously mixed. Feed-grade bentonite (BE), zeolite (ZE), and illite (IL) were sourced from commercial suppliers. For the purposes of Table 1, the reported Si and Al values reflect SiO_2_ and Al_2_O_3_ within the aluminosilicate lattice of these s and premix carriers; no soluble aluminum salts were added. Fish oil and deionized water (accounting for approximately 15% of the total mixture weight) were gradually added to achieve a consistent dough texture. Subsequently, the blended mixture was pelletized using a meat chopper (MN-22S, Hankook Fujee Industries, Hwaseong-si, Republic of Korea) equipped with a 1 mm die. The resulting pellets were dried in a convection dryer (LDO-150F, LabTech, Namyangju-si, Republic of Korea) at 25 °C for 24 h until the moisture content was reduced to below 9%. All diets were sealed in airtight containers and stored at −10 °C to maintain freshness and prevent nutrient degradation prior to use.

### 2.2. Feeding Trial

Juvenile Pacific white shrimp were obtained from Daesang Aquaculture Industry (Taean, Republic of Korea), a single commercial hatchery in Korea that maintains its own domesticated broodstock population used for routine seed production. The shrimp originated from one genetic stock, and no external lesions, abnormal behavior, or clinical signs of disease were observed at the time of collection. Immediately after procurement, the shrimp were transported to the dedicated shrimp research facility at Kunsan National University. Prior to the start of the feeding trial, all shrimp underwent a standardized two-week acclimation period, during which they were gradually transitioned from the hatchery diet to the basal experimental diet. After approximately three weeks of acclimation on a commercial diet supplied by Woosung Feed Co., Ltd. (Daejeon, Republic of Korea), individuals averaging 0.02 g were randomly assigned to 12 tanks (50 L each) at a density of 20 shrimp per tank, establishing three replicates per diet for the four test diets. The feeding trial was conducted for 9 weeks (63 days). During the experimental period, water temperature was maintained between 28.5 and 29.9 °C using submersible heaters, whereas dissolved oxygen levels (6.6–9.6 mg/L) and pH (6.8–7.4) were regulated using airstones and monitored physicochemical parameters with a YSI MultiLab 4010-3 water-quality meter (Yellow Springs, OH, USA). Illumination followed a 12:12 h light–dark cycle provided by fluorescent lighting. Restricted feeding, amounting to 6–14% of body weight per day, was administered in three feedings (09:00, 13:30, and 18:00) over a 9-week period. Additionally, 60% of the water in each tank was exchanged once a week to maintain optimal rearing conditions. Daily siphoning was performed to clear uneaten pellets and feces; an equal volume of seawater was then added to maintain tank volume.

### 2.3. Growth Performance and Biochemical Analyses

At the end of the feeding period, shrimp were fasted for 18 h to standardize physiological status before sampling, and the final body weight was measured at a predefined end-point. Growth performance was monitored by biweekly body-weight recordings, with the end-point weight used as the primary growth indicator. Performance indices were computed as follows:

$$Weight\;gain\left(WG,\%\right)=100\times \frac{\left(Wf-Wi\right)}{Wi}$$

$$Feed\;conversion\;ratio\left(FCR\right)=\frac{Dry\;feed\;intake\left(g\right)}{Wet\;weight\;gain\left(g\right)}$$

$$Specific\;growth\;rate\left(SGR,\%/day\right)=\left[\frac{\left(\ln Wf-\ln Wi\right)}{Days}\right]\times 100$$

$$Protein\;efficiency\;ratio\;(PER)=\frac{Wet\;weight\;gain\;\left(g\right)}{Total\;protein\;intake\;\left(g\right)}$$

$$Survival\;rate\;(\%)=\frac{Ni-Nm}{Ni}\times 100$$

*Wf* = final body weight; *Wi* = initial body weight; *Ni* = initial number; *Nm* = mortalities.

Biochemical analyses were conducted on eight randomly selected shrimp per replicate tank, which were anesthetized in ice-chilled water beforehand. Hemolymph was withdrawn from the ventral sinus using a 1 mL syringe preloaded with Alsever’s solution (Sigma-Aldrich, St. Louis, MO, USA). Samples were kept on ice and centrifuged at 5000× *g* for 10 min at 4 °C to obtain plasma, which was used immediately for biochemical and immune assays. Plasma activities of glutamic-oxaloacetic transaminase (GOT) and glutamic-pyruvate transaminase (GPT), which serve as biomarkers of hepatic function, were quantified using a FujiDRI-CHEM 3500i analyzer (Fuji Photo Film Ltd., Tokyo, Japan). All procedures adhered to ethical guidelines intended to minimize stress.

### 2.4. Proximate Analysis

Proximate analyses of the diets and whole-body shrimp were performed in triplicate to determine moisture, crude protein, crude lipid, and crude ash, following AOAC procedures [21,22]. Moisture is reported on a wet-weight basis, whereas crude protein, lipid, and ash are expressed on a dry-matter basis. Moisture content was determined gravimetrically according to AOAC. Approximately 2 g of homogenized sample was placed in pre-dried porcelain crucibles and dried in a forced-air oven (DO-150, Nexus, Republic of Korea) at 105 ± 2 °C until constant mass (typically 24 h). The result was calculated as the percentage mass loss relative to the initial wet weight. Total nitrogen was quantified by the Kjeldahl method in accordance with AOAC. Subsamples (0.5 g) were digested at 420 °C for 2 h in H_2_SO_4_ (98%) with a K_2_SO_4_:CuSO_4_ (10:1) catalyst using a digestion/distillation unit (Kjeltec 8400, FOSS, Hillerød, Denmark). Ammonia was distilled into 4% boric acid and titrated with 0.1 N HCl, and protein (%) was obtained from nitrogen using a conversion factor of 6.25. Crude lipid content was measured by Soxhlet extraction following AOAC. About 3 g of sample, wrapped in fat-free filter paper, was continuously extracted with anhydrous diethyl ether for 6 h on a Soxtec™ 8000 system (FOSS, Hillerød, Denmark). The solvent was evaporated at 60 °C, and lipids were quantified gravimetrically; values are expressed as % DM. Crude ash was determined by incineration as described by AOAC. Portions of 1 g were placed in pre-ashed crucibles and combusted at 550 ± 5 °C for 6 h in a muffle furnace (FHP-03, WiseTherm, Seoul, Republic of Korea). After cooling in a desiccator, ash content was expressed as the residual mass relative to the initial dry mass.

### 2.5. Amino Acid Composition Analysis

The amino acid composition of whole-body shrimp samples was determined by quantifying both the constituent and sulfur-containing amino acids using acid hydrolysis and performic acid oxidation, respectively. All analyses were performed using a SyKAM S433 Amino Acid Analyzer (SyKAM GmbH, Eresing, Germany) equipped with a post-column ninhydrin detection system. For constituent amino acids, approximately 0.02 g of the homogenized shrimp sample was hydrolyzed in 15 mL of 6 N HCl in a sealed glass tube at 115–135 °C for 18–24 h. The hydrolysate was evaporated to dryness under vacuum at 50 °C (130 rpm) with a concentrator. The residue was reconstituted with sample dilution buffer to a final volume of 25 mL, sonicated to ensure complete dissolution, and transferred to a 50 mL tube for analysis. For sulfur-containing amino acids (e.g., cysteine and methionine), approximately 0.02 g of sample was pre-oxidized with 20 mL of freshly prepared performic acid (a 9:1 mixture of 85% formic acid and 30% hydrogen peroxide) at 4 °C for 18–24 h. After oxidation, performic acid was evaporated under vacuum at 50 °C (130 rpm), and the residue was hydrolyzed in 15 mL of 6 N HCl in a sealed tube at 115–135 °C for 18–24 h. The hydrolysate was concentrated under vacuum, reconstituted in sample dilution buffer to 25 mL, sonicated, and transferred to a 50 mL tube. Both types of hydrolysates were analyzed using a SyKAM S433 Amino Acid Analyzer, and amino acid concentrations were determined by integrating chromatographic peak areas and comparing them with external standards using proprietary software.

### 2.6. Fatty Acid Composition Analysis

Total lipids were extracted from freeze-dried shrimp whole-body samples using a modified Folch method, in which diethyl ether was substituted for the original chloroform–methanol solvent system [22]. Briefly, 1 g of a freeze-dried sample was homogenized in 10 mL of diethyl ether. Subsequently, 2 mL of distilled water was added to induce phase separation. The mixture was centrifuged at 3000× *g* for 10 min and the upper ether phase containing the lipids was collected. The solvent was evaporated under a nitrogen stream, and the lipid extract was weighed and stored at −20 °C until further analysis. Fatty acid methyl esters (FAMEs) were prepared from the lipid extract using a modified transesterification protocol based on the AOAC method [21]. Approximately 25 mg of lipid was placed in a glass tube containing 1.5 mL of 0.5 N NaOH in methanol (prepared by dissolving 2 g NaOH in 100 mL of methanol). The tubes were flushed with nitrogen gas, sealed, vortexed, and heated at 100 °C for 6 min. After cooling, 2 mL of 14% boron trifluoride–methanol was added. The tubes were again flushed with nitrogen, vortexed, and heated at 100 °C for 30 min. After cooling to 30–40 °C, 1 mL of hexane was added, and the mixture was vortexed for 30 s. Saturated sodium chloride solution (5 mL of 36 g NaCl in 100 mL distilled water) was added and gently mixed to promote phase separation. The upper hexane layer containing the FAMEs was carefully collected using a Pasteur pipette, mixed with anhydrous sodium sulfate (Na_2_SO_4_) at a ratio of 1.5:1 (*w/w*, sodium sulfate to hexane) to remove residual moisture, vortexed, flushed with nitrogen, and stored at 4 °C until gas chromatographic analysis. FAMEs were analyzed using a Trace GC system (Trace GC, Theromo Finnigan, San Jose, CA, USA) equipped with an autosampler and a flame ionization detector (FID). Separation was achieved on a fused silica capillary column (007-CW-30-0.25F, Feries Silica, Quadrex Corporation, Woodbridge, CT, USA). The oven temperature was programmed as follows: initial temperature at 100 °C, increased to 220 °C at 5 °C/min, and subsequently increased to 240 °C at 3 °C/min. The injector and detector temperatures were maintained at 250 °C, while the carrier gas was supplied at a pressure of 65 psi. Instrument control and data acquisition were performed using Trace 2000 software, and specific parameters, such as injection volume, flow rates, and autosampler washing cycles, were optimized for reproducibility. Prior to analysis, the system was equilibrated for 10–20 min and the FID flame was ignited. Fatty acids were identified by comparing their retention times with those of certified FAME standards.

### 2.7. Immunological and Antioxidant Parameters

Immunological and antioxidant parameters in shrimp were evaluated using plasma isolated from hemolymph samples, which were centrifuged at 3000× *g* for 10 min at 4 °C. Phagocytic activity was quantified by incubating plasma with a nitro blue tetrazolium (NBT) solution, which was reduced to formazan and measured spectrophotometrically at 540 nm. Lysozyme activity was determined by measuring the rate of lysis of a *Micrococcus lysodeikticus* suspension in phosphate buffer (pH 6.2), with absorbance changes recorded at 540 nm over a 5 min period. Phenoloxidase (PO) activity was measured by activating plasma with α-chymotrypsin, followed by incubation with L-3,4-dihydroxyphenylalanine (L-DOPA) as a substrate, and tracking the increase in absorbance at 490 nm over 30 min. Antioxidant enzyme activities were also evaluated: superoxide dismutase (SOD) was quantified using a commercial assay kit (Sigma-Aldrich, 19160, St. Louis, MO, USA) based on the inhibition of formazan dye formation; glutathione peroxidase (GPx) activity was measured using assay kits (BioVision, Inc., Milpitas, CA, USA), with GPx activity determined by the rate of NADPH oxidation at 340 nm. Antiprotease activity was analyzed by incubating the plasma with trypsin and azocasein and measuring the residual protease activity at 366 nm to calculate the percentage inhibition relative to the controls. All measurements were performed in triplicate and normalized to the total protein content determined via the Bradford method [23]. Appropriate blanks and standards were included in all measurements to ensure assay accuracy and reproducibility.

### 2.8. Digestibility Test

Apparent digestibility coefficients (ADCs) were determined using shrimp (4.05 ± 0.48 g) rather than the 0.02 g shrimp used in the growth trial. Extremely small shrimp do not produce sufficient or stable fecal material, and feces cannot be clearly distinguished from uneaten feed, making marker-based digestibility measurements technically impractical. Therefore, individuals were grown to approximately 4 g to allow reliable fecal recovery and reduce sampling errors associated with fecal disintegration. To determine apparent digestibility, we added 1.0% chromium(III) oxide (Cr_2_O_3_; Sigma-Aldrich, St. Louis, MO, USA) to all test diets as an inert indicator. Shrimp were randomly distributed to three acrylic tanks (110 L each) at a density of 15 individuals per tank and offered the diets two times per day at 08:30 and 14:00 over six weeks. Uneaten feed and tank debris were removed via siphoning 1 h after each feeding to maintain the water quality. Fecal material was collected from each tank twice daily at 13:30 and 18:30 via gentle siphoning to minimize sample loss or contamination. The collected feces were immediately frozen at −80 °C and subsequently freeze-dried for analysis. Apparent digestibility coefficients (ADCs) for dry matter and protein were calculated using the following formulae: ADC for dry matter (%) = 100 − 100 × (% Cr_2_O_3_ in diet/% Cr_2_O_3_ in feces); ADC for protein (%) = 100 − 100 × (% Cr_2_O_3_ in diet/% Cr_2_O_3_ in feces) × (% protein in feces/% protein in diet). Dietary and fecal samples were acid-digested and analyzed for chromium using atomic absorption spectroscopy. Crude protein and dry matter contents were analyzed according to standard proximate analysis procedures. All analyses were performed in triplicate to ensure accuracy and reproducibility.

### 2.9. Statistical Analyses

All data were analyzed by one-way analysis of variance, followed by Tukey’s HSD test using SPSS Statistics for Windows (version 26.0; IBM Corp., Armonk, NY, USA). Statistical significance was set at a probability level of *p* < 0.05. All data were reported as mean ± SD. Proportional data were arcsine transformed prior to statistical analysis to ensure normality and homogeneity of variance.

## 3. Results

### 3.1. Growth Performance and Carcass Composition

Growth performance for *L. vannamei* across the four dietary treatments (CON, BE, ZE, IL) is presented in Table 2. All groups started the experiment with an initial mean body weight of 0.02 ± 0.001 g, for which no significant differences were detected among treatments (*p* > 0.05). At the end of the feeding trial, final body weight was significantly higher in shrimp specimens fed the IL diet (5.95 ± 0.97 g) than that in the ZE (5.60 ± 0.90 g), BE (5.20 ± 0.96 g), and CON (4.95 ± 0.99 g) diets (*p* < 0.05). Weight gain (WG) followed a similar trend, with shrimp in the IL group achieving the highest WG (29,624 ± 820%), which was significantly higher than that in the ZE (27,918 ± 396%), BE (25,924 ± 1070%), and CON (24,702 ± 660%) groups (*p* < 0.05). The FCR was significantly improved in the IL (1.10 ± 0.03) and ZE (1.16 ± 0.02) groups than that in the BE (1.26 ± 0.05) and CON (1.32 ± 0.04) groups (*p* < 0.05), thereby indicating enhanced feed utilization efficiency. SGR increased significantly across diets, with the highest SGR observed in shrimp specimens fed IL (9.04 ± 0.04%/day), followed by ZE (8.95 ± 0.02%/day), BE (8.83 ± 0.07%/day), and CON (8.75 ± 0.04%/day) (*p* < 0.05). Similarly, the PER was significantly greater in the IL group (2.59 ± 0.07) than that in the ZE (2.45 ± 0.03), BE (2.26 ± 0.09), and CON (2.17 ± 0.06) groups (*p* < 0.05). Survival rates ranged from 91.7% to 93.3%, with no significant differences observed among the treatments (*p* > 0.05), thereby suggesting that all diets were equally effective in supporting shrimp health and survival under experimental conditions.

The whole-body proximate composition of *L. vannamei* fed the experimental diets is presented in Table 3. The moisture content ranged from 76.49% to 78.76%, with no significant differences among the treatments (*p* > 0.05). Shrimp specimens fed the ZE (17.76 ± 0.18%) and IL (17.52 ± 0.11%) diets exhibited significantly higher crude protein content than those fed the BE (16.25 ± 0.21%) and CON (15.80 ± 0.19%) diets (*p* < 0.05). This suggests that dietary supplementation with ZE and IL contributes to enhanced protein retention in shrimp. Crude lipid content was highest in shrimp specimens fed the ZE group (1.27 ± 0.38%), although differences among treatments were not statistically significant (*p* > 0.05). Crude ash content remained consistent across all dietary groups, with no significant variations observed (*p* > 0.05). The whole-body amino acid and fatty acid composition of *L. vannamei* fed the experimental diets is shown in Table 4 and Table 5. The results revealed no statistically significant diet effects on whole-body amino acids or fatty acids (*p* > 0.05). For amino acids, glutamic acid was the most abundant across treatments (8.85–9.19%), and essential amino acids such as lysine, leucine, methionine, and histidine showed narrow, non-significant variation among groups (e.g., lysine 4.50–4.57% in IL–ZE). For fatty acids, palmitic acid (C16:0) was the predominant SFA (~18–19% of total FAs), oleic acid (C18:1n9) was the major MUFA (22.7–24.9%), and omega-3 PUFAs were consistently represented (DHA 12.2–14.0%, EPA 7.7–8.8%) regardless of diet. Sums of SFA, MUFA, and PUFA remained comparable among treatments (SFA 31.46–32.67%, MUFA 24.72–27.40%, PUFA 32.94–39.43%; all *p* > 0.05). Collectively, these data indicate that the iso-N/iso-E diets maintained whole-body AA/FA homeostasis, and between-diet performance differences were not accompanied by significant shifts in tissue AA/FA profiles.

### 3.2. Biochemical and Immunological Parameters

**Figure 1:**
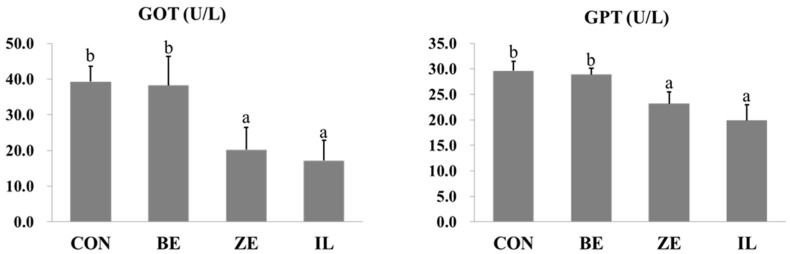
Glutamate-oxalacetate transaminase (GOT) and glutamate-pyruvate transaminase (GPT) activities of Pacific white shrimp (*Litopenaeus vannamei*) specimens fed experimental diets. Different superscript letters (a–b) indicate significant differences among treatments (*p* < 0.05; one-way ANOVA, Tukey’s HSD test).

The activities of glutamate-oxalacetate transaminase (GOT) and glutamate-pyruvate transaminase (GPT) in the hemolymph of *L. vannamei* are shown in Figure 1. The activities of both enzymes were significantly different (*p* < 0.05) among the dietary treatments. Shrimp specimens fed ZE and IL diets exhibited significantly lower GOT levels (approximately 20 and 15 U/L, respectively) than those fed the CON and BE diets (both approximately 40 U/L). A similar trend was observed for GPT activity, where the values in the ZE and IL groups were significantly reduced (approximately 22 U/L and 20 U/L, respectively), in contrast to the elevated levels observed in the CON and BE groups (approximately 30 U/L).

The non-specific immune parameters and antioxidant status of shrimp specimens fed experimental diets (CON, BE, ZE, and IL) are presented in Table 6. Significant differences (*p* < 0.05) were observed among the dietary treatments for most immune and antioxidant indicators. Nitroblue tetrazolium (NBT) activity, an indicator of respiratory burst activity, was significantly higher in shrimp specimens fed the ZE (2.23 ± 0.11) and IL (2.34 ± 0.07) diets than those fed the BE diet (1.95 ± 0.07), with intermediate values in the CON group (2.11 ± 0.04). PO activity also followed a similar trend, with the highest value recorded in the IL group (0.178 ± 0.012), thereby indicating enhanced prophenoloxidase cascade activation. GPx activity, reflecting the antioxidant capacity of shrimp, was significantly higher in the IL group (31.83 ± 1.23) than that in the CON and BE groups (24.47 ± 3.25 and 24.73 ± 2.11, respectively), thereby suggesting improved oxidative stress defense. The ZE group showed intermediate GPx values (27.87 ± 1.63), which were statistically different from the IL and CON groups. Lysozyme activity, a key humoral immune component, was significantly higher in the IL group (4.20 ± 0.08) than that in the other groups, all of which remained statistically similar (ranging from 3.78 to 3.84). In contrast, the antiprotease activity did not differ significantly among the treatments, indicating a stable baseline protease inhibitory function.

### 3.3. Apparent Digestibility Coefficients (ADC)

The ADCs of dry matter and crude protein for *L. vannamei* under the experimental diets are summarized in Table 7. Although no significant differences were observed in the apparent dry matter digestibility (ADCd), the values ranged from 84.3 ± 1.26% in the CON group to 89.0 ± 1.69% in the IL group, thereby indicating a numerically higher digestibility in the IL and ZE diets. In contrast, significant differences (*p* < 0.05) were found in the apparent crude protein digestibility (ADCp). Shrimp specimens fed the IL diet exhibited the highest ADCp value (93.3 ± 0.70%), which was significantly higher than the values recorded in the CON (87.3 ± 0.92%), BE (87.8 ± 0.88%), and ZE (89.1 ± 1.11%) groups.

## 4. Discussion

The findings of the study clearly demonstrate that dietary supplementation with silicate minerals, particularly IL, can substantially improve physiological and nutritional performance in *L. vannamei*. Shrimp specimens fed the IL-supplemented diet (IL group) consistently exhibited superior outcomes compared to those fed the control (CON), ZE-supplemented, and BE-supplemented diets. The application of silicate minerals in aquaculture has garnered increased attention because of their potential to enhance the growth, immunity, and physiological stability across various aquatic species. Previous studies involving African catfish (*Clarias gariepinus*) reported that the inclusion of a montmorillonite–IL/muscovite blend at 0.5% in commercial diets contributed to improved growth and reduced body size variation, along with a marked reduction in external skin lesions, although no statistical significance was reported in performance metrics [20]. These findings highlight that silicates may enhance surface health and welfare in cultured fish species without adversely affecting organ function or systemic metabolism. Bentonite (BE), despite its widespread use as an aluminosilicate mineral in aquaculture and terrestrial livestock, has demonstrated highly variable efficacy depending on species, environmental conditions, and application context. In juvenile European sea bass (*Dicentrarchus labrax*), dietary or environmental supplementation with BE has been associated with improved water quality, reduced ammonia accumulation, enhanced immune responses, and modest improvements in growth performance under certain conditions [24]. However, in the present study involving *L. vannamei*, dietary BE supplementation did not significantly influence growth performance, hematological indices, or digestive physiology. A key factor contributing to these discrepancies is the experimental environment under which BE has been tested in previous research. Several studies reporting strong benefits of BE were conducted under toxin-challenge or chemically stressful conditions, where the mineral’s adsorptive and detoxifying capacities are physiologically meaningful [9,10,25,26]. For example, Yadav et al. demonstrated that 2–4% dietary BE effectively mitigated feed-borne iron toxicity in *Oncorhynchus mykiss*, restoring hepatic integrity and normalizing hematological profiles [9]. Similarly, Neeratanaphan and Tengjaroenkul found that 1% BE significantly alleviated aflatoxin B1 toxicity in *L. vannamei*, improving survival, growth, and hepatopancreatic condition [26]. By contrast, studies conducted under non-stressful and toxin-free conditions often show minimal or inconsistent effects, suggesting that BE’s functional roles may be context-dependent rather than universally beneficial. El-Dahhar et al. reported performance improvements and reduced ammonia excretion in *D. labrax* at 0.5–1% BE, yet other investigations noted negligible growth-enhancing effects when basal diets were nutritionally adequate and free of contaminants [8]. Taken together, the collective evidence indicates that physiological contributions of BE are most pronounced under stress- or toxin-induced conditions, such as mycotoxin exposure or oxidative challenge, where its adsorption capacity and gut-protective properties are likely to be engaged. Under the stable water quality, pathogen-free conditions, and toxin-free diets used in our experiment, such mechanisms may not have been activated, which provides a plausible explanation for the absence of significant BE-related effects in this study. In the present study, dietary IL supplementation yielded modest but consistent improvements across multiple performance indicators, including growth efficiency, feed utilization, antioxidant response, and hepatopancreatic conditions. Although these outcomes support the functional role of IL in shrimp nutrition, these results should be interpreted within the broader context of mineral bioactivity. Rather than asserting universal superiority, these findings indicate the potential of IL as a viable dietary component under specific rearing conditions. Ultimately, the effectiveness of mineral additives in aquaculture is shaped by multiple interacting factors, including the biological characteristics of the species, physicochemical properties of the mineral, environmental conditions, and intended functional objective (e.g., toxin binding vs. nutrient enhancement). This highlights the importance of conducting species-specific evaluations when selecting functional additives for aquafeed formulations. According to previous studies on illite supplementation, the observed growth advantage most plausibly reflects aluminosilicate-mediated adsorption and ion exchange that sequester luminal toxins (e.g., mycotoxins and ammonium) and moderate ionic conditions, together with mucosal support that enhances digestive capacity and apparent protein digestibility, thereby stabilizing the gastrointestinal milieu and lowering metabolic costs [27]. These mechanisms remain hypothesis-generating and require targeted follow-up studies for confirmation.

This study aimed to compare the physiological effects of various IL-based dietary supplements, with a particular focus on the influence of IL on the hepatopancreatic function, antioxidant status and immune response in shrimp. The results revealed that shrimp specimens fed the IL-supplemented diet exhibited significantly reduced hemolymph levels of GOT and GPT, both of which are commonly used biomarkers of hepatic stress and cellular injury. The levels of these enzymes are often elevated in aquatic species exposed to dietary toxins or environmental pollutants, suggesting that mineral-based additives may confer hepatoprotective effects under these conditions. Consistent with these findings, Abbas et al. [28] reported that dietary BE supplementation in Nile tilapia effectively mitigated diazinon-induced hepatic and renal damage by restoring antioxidant enzyme activities and normalizing hemolymph biochemical parameters. Similarly, IL supplementation in the present study significantly increased GPx activity, indicating an enhanced antioxidant defense system capable of neutralizing reactive oxygen species and preserving cellular integrity. In addition to improving liver health, silicate minerals have demonstrated positive effects on innate immune responses. In this study, shrimp receiving an IL-enriched diet showed elevated NBT, PO, and lysozyme activities, which are key indicators of respiratory burst, melanization pathway activation, and antibacterial defense, respectively. These findings align with those of Xu et al. [29], who reported improved growth performance, immune enzyme activity, and disease resistance in largemouth bass (*Micropterus salmoides*) following supplementation with azomite, a natural trace mineral complex. The antimicrobial potential of azomite-based composites has been highlighted in other studies. Saljoghi et al. [30] demonstrated that a chitosan–BE composite exhibited strong antibacterial activity against *Aeromonas hydrophila*, a common aquaculture pathogen, suggesting that minerals may exert adsorptive, immunostimulatory, and antimicrobial effects. Karimi et al. [31] reported that montmorillonite supplementation in rainbow trout (*Oncorhynchus mykiss*) enhanced both growth performance and immune-related gene expression, underscoring the broader physiological roles of mineral additives beyond toxin binding. Collectively, these findings indicate that various minerals, including IL, contribute to improved immune competence and physiological resilience in aquaculture species via multiple mechanisms. Transaminase activities were lower in the IL group, which is consistent with a more favorable hepatic status. Because histological coverage was limited, this finding should be interpreted cautiously, and broader liver histology is needed to confirm hepatoprotection.

In the present study, shrimp fed an IL-supplemented diet exhibited significantly enhanced crude protein digestibility and retention. These findings are similar to those observed in previous studies utilizing montmorillonite, another aluminosilicate, in which improvements in intestinal morphology and digestive enzyme activity were associated with improved feed efficiency and growth rates in juvenile turbots [32]. These enhancements may be attributed to the high adsorptive capacity and structural stability of, which promote nutrient uptake and may help regulate the gut microbiota, thereby improving the overall metabolic efficiency. However, not all minerals have the same nutritional effects. For example, the inclusion of 10–20% natural ZE in the diets of European sea bass did significantly change protein digestibility or growth performance [33]. Instead, the observed reduction in feed efficiency reported in some studies appears to be primarily attributable to nutrient dilution rather than a direct negative effect of the mineral itself [33]. This emphasizes the importance of both mineral type and inclusion level when evaluating the functional benefits of dietary additives. In the present study, whole-body amino-acid profiles did not differ significantly among diets; therefore, we do not ascribe any changes in essential amino acids to illite. Possible mechanisms for illite’s effects remain hypothesis-generating—for example, naturally occurring trace minerals in illite (e.g., K, Mg, Fe) could support enzymatic processes related to ATP production, protein synthesis, or antioxidant defenses—but these were not directly measured here and should be tested in follow-up trials. Consistent with this view, previous research in growing–finishing pigs reported improved dry-matter digestibility and growth performance with dietary illite, suggesting enhanced nutrient utilization as a plausible pathway [15]. While bentonite, zeolite, and illite are known to exhibit properties such as toxin adsorption, modulation of gastrointestinal microbiota, and improvement of gut environmental stability, these mechanisms were not directly evaluated in the present study. Therefore, such interpretations should be regarded as hypotheses rather than confirmed mechanisms. The physiological responses observed here—such as enhanced immune parameters and antioxidant activity—may be partly related to these functional properties, but the underlying pathways require further verification through histological assessment, microbiome profiling, and toxin-binding assays. Future studies incorporating these approaches will be essential to validate the mechanistic roles of mineral supplements in shrimp.

Although clay minerals frequently improve growth, feed efficiency, and water quality, their effects are dose-dependent and may reverse beyond an optimal range [8,34]. In practical feeds, one key mechanism of adverse response at high inclusion is nutrient dilution when high-ash minerals replace nutrient-dense ingredients; therefore, maintaining iso-nitrogenous/iso-energetic formulation and conservative inclusion is essential [34]. Across aquaculture, effective feed-additive ranges are commonly reported around ~0.4–4.5% depending on mineral type and species, zeolite can redistribute heavy metals across tissues (e.g., lowering Pb in muscle but increasing it in kidney), underscoring the need to monitor tissue-specific burdens during long-term use [28,35]. Safety is generally favorable within recommended ranges, but pathology at very high bentonite inclusion has been documented, reinforcing conservative dosing and species-specific validation [32]. Finally, the literature heterogeneity (species, systems, and statistical rigor) warrants cautious generalization and motivates commercial-scale, species-targeted trials to refine inclusion ceilings and verify sustainability benefits [36]. We did not measure effluent or sediment endpoints in this trial. Future work will incorporate mass-balance sampling of water and sludge (TSS, TAN/NH_3_, PO_4_-P), standard leaching assays for trace metals, and benthic community assessments in tank/pond mesocosms to quantify environmental fate and risk.

Overall, our findings indicate that illite supplementation supports not only protein metabolism but also broader physiological functions, including gut performance and mineral bioavailability. These results are consistent with reports from both aquatic and terrestrial species and highlight the promise of illite as a versatile functional feed additive across production systems. While the dataset integrates growth, compositional, digestibility, and innate immune/antioxidant endpoints, additional mechanistic evidence would further strengthen these conclusions. Follow-up studies should expand liver histology and include targeted assays of digestive enzymes and related pathways to substantiate the inferences drawn here.

## 5. Conclusions

This study demonstrates that illite exerted the strongest and most consistent effects among the evaluated silicate minerals, improving growth performance, feed and protein utilization, hepatopancreatic function, and innate immune and antioxidant responses in *L. vannamei*. These collective outcomes substantiate the suitability of illite as a functional mineral additive in shrimp aquafeeds. Nevertheless, the present work did not assess mineral or trace-element residues in shrimp tissues, nor did it quantify environmental processes such as leaching or effluent loading; consequently, the long-term fate of dietary minerals remains to be clarified. Furthermore, the mechanistic basis underlying the observed physiological responses—whether through luminal adsorption, modulation of gut microbiota, or effects on mucosal integrity—requires targeted verification. Future investigations should therefore incorporate dose–response frameworks, tissue-residue profiling, and environmental assessments under farm-scale conditions, together with mechanistic studies employing histology, microbiome sequencing, metabolomics, and toxin-binding assays. These efforts will be essential for establishing safe, effective, and ecologically robust applications of illite in commercial shrimp production.

## Figures and Tables

**Table 1:** Diet formulation and proximate analysis of four test diets for *Litopenaeus vannamei* (DM basis, %).

Ingredients (%)	Experimental Diets			
	CON	BE	ZE	IL
Tuna by product meal ^a^	40.0	40.0	40.0	40.0
Squid liver powder ^a^	20.0	20.0	20.0	20.0
Soybean meal ^a^	15.0	15.0	15.0	15.0
Wheat flour ^a^	15.0	15.0	15.0	15.0
Starch ^a^	5.0	0	0	0
Fish oil ^b^	3.0	3.0	3.0	3.0
Vitamin and mineral mix ^c^	2.0	2.0	2.0	2.0
Bentonite ^a^	0	5.0	0	0
Zeolite ^a^	0	0	5.0	0
Illite ^d^	0	0	0	5.0
Chemical composition (%, dry mater)				
Moisture	8.74	8.70	8.84	8.72
Crude protein	35.04	35.35	35.09	35.21
Crude lipid	9.63	9.75	9.72	9.76
Crude ash	7.87	9.98	9.76	9.10

^a^ The Feed, Goyang, Republic of Korea. ^b^ Ewha Oil & Fat Industrial Co., Ltd., Busan, Republic of Korea. ^c^ Contains (as mg kg^−1^ in diets): Ascorbic acid, 300; dl-Calcium pantothenate, 150; Choline bitate, 3000; Inositol, 150; Menadion, 6; Niacin, 150; Pyridoxine⋅HCl, 15; Rivoflavin, 30; Thiamine mononitrate, 15; dl-α-Tocopherol acetate, 201; Retinyl acetate, 6; Biotin, 1.5; Folic acid, 5.4; Cobalamin, 0.06; NaCl, 437.4; MgSO_4_ ·7H_2_O, 1379.8; ZnSO_4_ ·7H_2_O, 226.4; Fe-Citrate, 299; MnSO_4_, 0.016; FeSO_4_, 0.0378; CuSO_4_, 0.00033; Calciumiodate, 0.0006; MgO, 0.00135; NaSeO_3_, 0.00025. ^d^ Supplied by Illite village Co., Ltd., Yeongdong-gun, Republic of Korea.

**Table 2:** Growth performance of *Litopenaeus vannamei* during the 9-week trial (CON, BE, ZE, IL) ^1^.

	CON	BE	ZE	IL
Initial mean weight (g)	0.020 ± 0.001	0.020 ± 0.001	0.020 ± 0.001	0.020 ± 0.001
Final mean weight (g)	4.95 ± 0.99 ^c^	5.20 ± 0.96 ^c^	5.60 ± 0.90 ^b^	5.95 ± 0.97 ^a^
Weight gain (%)	24,702 ± 660 ^c^	25,924 ± 1070 ^c^	27,918 ± 396 ^b^	29,624 ± 820 ^a^
Feed conversion ratio	1.32 ± 0.04 ^b^	1.26 ± 0.05 ^b^	1.16 ± 0.02 ^a^	1.10 ± 0.03 ^a^
Specific growth rate (%/day)	8.75 ± 0.04 ^c^	8.83 ± 0.07 ^c^	8.95 ± 0.02 ^b^	9.04 ± 0.04 ^a^
Protein efficiency ratio	2.17 ± 0.06 ^c^	2.26 ± 0.09 ^c^	2.45 ± 0.03 ^b^	2.59 ± 0.07 ^a^
Survival rate (%)	93.3 ± 2.9	91.7 ± 5.8	91.7 ± 2.9	93.3 ± 2.9

^1^ Values are mean ± SD (n = 3 tanks/treatment). Different superscript letters within a row indicate *p* < 0.05 (one-way ANOVA, Tukey’s HSD).

**Table 3:** Whole-body proximate composition of *Litopenaeus vannamei* after the 9-week feeding trial (CON, BE, ZE, IL; DM basis, %) ^1^.

	**CON**	**BE**	**ZE**	**IL**
Moisture (%)	78.76 ± 2.13	78.18 ± 1.54	76.49 ± 1.16	76.74 ± 0.95
Crude protein (%)	15.80 ± 0.19 ^b^	16.25 ± 0.21 ^b^	17.76 ± 0.18 ^a^	17.52 ± 0.11 ^a^
Crude lipid (%)	0.88 ± 0.04	0.78 ± 0.05	1.27 ± 0.38	1.04 ± 0.13
Crude ash (%)	3.21 ± 0.14	3.32 ± 0.24	3.18 ± 0.43	3.22 ± 0.17

^1^ Values are mean ± SD (n = 3 tanks/treatment). Different superscript letters within a row indicate *p* < 0.05 (one-way ANOVA, Tukey’s HSD).

**Table 4:** Total amino acid profile in whole-body *Litopenaeus vannamei* at the end of the 9-week trial (CON, BE, ZE, IL; DM basis, %) ^1^.

	CON	BE	ZE	IL
**Essential amino acids (EAAs)**				
Arginine	3.94 ± 0.18	3.85 ± 0.17	4.05 ± 0.20	3.90 ± 0.15
Threonine	2.44 ± 0.12	2.42 ± 0.10	2.55 ± 0.14	2.50 ± 0.13
Valine	3.16 ± 0.10	3.13 ± 0.11	3.27 ± 0.15	3.23 ± 0.10
Isoleucine	2.68 ± 0.07	2.65 ± 0.15	2.79 ± 0.11	2.73 ± 0.09
Leucine	4.34 ± 0.04	4.32 ± 0.08	4.55 ± 0.12	4.49 ± 0.05
Methionine	1.26 ± 0.09	1.25 ± 0.07	1.38 ± 0.08	1.34 ± 0.09
Lysine	4.31 ± 0.04	4.36 ± 0.02	4.57 ± 0.03	4.50 ± 0.06
Phenylalanine	2.76 ± 0.12	2.73 ± 0.13	2.90 ± 0.16	2.84 ± 0.12
Histidine	1.28 ± 0.12	1.27 ± 0.10	1.44 ± 0.09	1.33 ± 0.10
**Non-essential amino acids (NEAAs)**				
Serine	2.17 ± 0.09	2.10 ± 0.20	2.13 ± 0.10	2.08 ± 0.09
Glutamic acid	8.85 ± 0.23	8.88 ± 0.14	9.19 ± 0.21	9.14 ± 0.32
Proline	3.29 ± 0.11	3.14 ± 0.15	3.81 ± 0.13	3.55 ± 0.13
Glycine	5.76 ± 0.13	5.78 ± 0.11	5.27 ± 0.11	5.55 ± 0.23
Alanine	4.40 ± 0.11	4.32 ± 0.13	4.67 ± 0.11	4.63 ± 0.14
Tyrosine	2.25 ± 0.10	2.13 ± 0.12	1.93 ± 0.14	1.87 ± 0.13
Aspartic acid	5.97 ± 0.29	5.91 ± 0.12	6.16 ± 0.11	5.95 ± 0.12
Cysteine	0.48 ± 0.08	0.47 ± 0.06	0.54 ± 0.08	0.50 ± 0.04

^1^ Values are mean ± SD (n = 3 tanks/treatment).

**Table 5:** Whole-body fatty acid composition of *Litopenaeus vannamei* after the 9-week feeding trial (CON, BE, ZE, IL; DM basis, %) ^1^.

	CON	BE	ZE	IL
C16:0	18.27 ± 0.45	18.79 ± 0.28	18.94 ± 0.62	18.73 ± 0.35
C16:1	1.42 ± 0.23	1.49 ± 0.19	1.61 ± 0.18	1.42 ± 0.20
C17:0	1.18 ± 0.13	1.26 ± 0.15	1.14 ± 0.11	1.24 ± 0.17
C18:0	9.18 ± 0.42	9.81 ± 0.38	8.89 ± 0.43	9.35 ± 0.28
C18:1n9	22.68 ± 1.02	22.85 ± 1.35	24.93 ± 2.06	23.00 ± 2.10
C18:2n6	12.60 ± 0.85	11.99 ± 0.63	12.16 ± 1.01	12.25 ± 0.44
C20:1n9	2.04 ± 0.23	1.98± 0.17	2.47 ± 0.25	2.10 ± 0.22
C20:2	1.41 ± 0.15	1.32 ± 0.16	1.44 ± 0.12	1.38 ± 0.09
C20:5n3 (EPA)	8.47 ± 0.39	8.79 ± 0.29	7.73 ± 0.43	8.46 ± 0.41
C22:6n3 (DHA)	14.04 ± 1.12	12.16 ± 1.80	13.46 ± 1.32	13.59 ± 0.98
SFA ^2^	31.46 ± 4.84	32.67 ± 4.05	32.02 ± 3.18	32.67 ± 3.85
MUFA ^3^	24.72 ± 2.98	24.83 ± 1.85	24.40 ± 2.98	24.83 ± 4.07
PUFA ^4^	39.43 ± 3.71	38.05 ± 3.55	36.69 ± 4.42	38.05 ± 3.23
Unknown fatty acid ^5^	8.72 ± 0.95	5.11 ± 0.56	7.23 ± 0.48	8.48 ± 1.11

^1^ Values are mean ± SD (n = 3 tanks/treatment). ^2^ Saturated fatty acid. ^3^ Monounsaturated fatty acid. ^4^ Polyunsaturated fatty acid. ^5^ Unknown fatty acids represent minor chromatographic peaks that could not be matched with available GC–FID reference standards.

**Table 6:** Non-specific immune and antioxidant parameters of *Litopenaeus vannamei* fed the CON, BE, ZE, and IL diets ^1^.

	CON	BE	ZE	IL
NBT ^2^	2.11 ± 0.04 ^ab^	1.95 ± 0.07 ^b^	2.23 ± 0.11 ^a^	2.34 ± 0.07 ^a^
Lysozyme (U mL^−1^) ^3^	3.81 ± 0.15 ^b^	3.84 ± 0.11 ^b^	3.78 ± 0.15 ^b^	4.20 ± 0.08 ^a^
Antiprotease ^4^	18.87 ± 0.55	18.67 ± 0.76	18.33 ± 0.86	18.90 ± 0.61
PO ^5^	0.147 ± 0.006 ^ab^	0.144 ± 0.014 ^b^	0.155 ± 0.012 ^ab^	0.178 ± 0.012 ^a^
GPx (mU mL^−1^) ^6^	24.47 ± 3.25 ^b^	24.73 ± 2.11 ^b^	27.87 ± 1.63 ^ab^	31.83 ± 1.23 ^a^
SOD (U mL^−1^) ^7^	52.80 ± 6.47	52.17 ± 3.27	55.53 ± 5.87	52.97 ± 2.57

^1^ Values are mean ± SD (n = 3 tanks/treatment). Different superscript letters within a row indicate *p* < 0.05 (one-way ANOVA, Tukey’s HSD). ^2^ NBT: Nitroblue tetrazolium activity (absorbance). ^3^ Lysozyme activity (U mL^−1^). ^4^ Antiprotease activity (% inhibition). ^5^ PO: Phenoloxidase activity (absorbance). ^6^ GPx: Glutathione peroxidase activity (mU mL^−1^). ^7^ SOD: Superoxide dismutase (U mL^−1^).

**Table 7:** Apparent digestibility coefficients (ADCs) for dry matter (ADCd) and crude protein (ADCp) in *Litopenaeus vannamei* fed the CON, BE, ZE, and IL diets ^1^.

	CON	BE	ZE	IL
ADCd (%) ^2^	84.3 ± 1.26	85.2 ± 1.22	86.8 ± 1.20	89.0 ± 1.69
ADCp (%) ^3^	87.3 ± 0.92 ^b^	87.8 ± 0.88 ^b^	89.1 ± 1.11 ^b^	93.3 ± 0.70 ^a^

^1^ Values are mean ± SD (n = 3 tanks/treatment). Different superscript letters within a row indicate *p* < 0.05 (one-way ANOVA, Tukey’s HSD). ^2^ ADCd = apparent digestibility coefficient of dry matter (%). ^3^ ADCp = apparent digestibility coefficient of crude protein (%).

## Data Availability

Data are contained within the article.
